# Transfer Effects to a Multimodal Dual-Task after Working Memory Training and Associated Neural Correlates in Older Adults – A Pilot Study

**DOI:** 10.3389/fnhum.2017.00085

**Published:** 2017-02-24

**Authors:** Stephan Heinzel, Jérôme Rimpel, Christine Stelzel, Michael A. Rapp

**Affiliations:** ^1^Clinical Psychology and Psychotherapy, Freie Universität BerlinBerlin, Germany; ^2^Social and Preventive Medicine, University of PotsdamPotsdam, Germany; ^3^Department of Psychology, Humboldt-Universität zu BerlinBerlin, Germany; ^4^Clinical Psychology and Neuropsychology, Johannes Gutenberg University MainzMainz, Germany; ^5^International Psychoanalytic UniversityBerlin, Germany; ^6^Berlin School of Mind and BrainBerlin, Germany

**Keywords:** working memory, cognitive training, modality, dual-task, aging, transfer, fMRI, neuroimaging

## Abstract

Working memory (WM) performance declines with age. However, several studies have shown that WM training may lead to performance increases not only in the trained task, but also in untrained cognitive transfer tasks. It has been suggested that transfer effects occur if training task and transfer task share specific processing components that are supposedly processed in the same brain areas. In the current study, we investigated whether single-task WM training and training-related alterations in neural activity might support performance in a dual-task setting, thus assessing transfer effects to higher-order control processes in the context of dual-task coordination. A sample of older adults (age 60–72) was assigned to either a training or control group. The training group participated in 12 sessions of an adaptive n-back training. At pre and post-measurement, a multimodal dual-task was performed in all participants to assess transfer effects. This task consisted of two simultaneous delayed match to sample WM tasks using two different stimulus modalities (visual and auditory) that were performed either in isolation (single-task) or in conjunction (dual-task). A subgroup also participated in functional magnetic resonance imaging (fMRI) during the performance of the n-back task before and after training. While no transfer to single-task performance was found, dual-task costs in both the visual modality (*p* < 0.05) and the auditory modality (*p* < 0.05) decreased at post-measurement in the training but not in the control group. In the fMRI subgroup of the training participants, neural activity changes in left dorsolateral prefrontal cortex (DLPFC) during one-back predicted post-training auditory dual-task costs, while neural activity changes in right DLPFC during three-back predicted visual dual-task costs. Results might indicate an improvement in central executive processing that could facilitate both WM and dual-task coordination.

## Introduction

Aging is associated with neurochemical, structural, and functional brain changes (Grady, 2012) that affect various cognitive functions. One central cognitive function which is affected by these changes and known to be declined in older age is working memory (WM) (Bopp and Verhaeghen, 2005). Neuroimaging studies have identified brain areas that play a key role in WM processing including lateral prefrontal cortex, inferior parietal lobule, as well as medial frontal regions (Miller and Cohen, 2001; Owen et al., 2005; D’Esposito, 2007). It has been suggested that efficient functioning of such a fronto-parietal WM network is reduced in older adults, as indicated by relatively higher activation at low WM load and relatively lower activation at high WM load when compared to younger adults (Schneider-Garces et al., 2010; Nagel et al., 2011; Heinzel et al., 2014a). These age-related changes in WM load-dependent activation patterns have been described within the framework of the compensation-related utilization of neural circuits hypothesis (CRUNCH, Reuter-Lorenz and Cappell, 2008). Specifically, an over-recruitment of neural resources at low WM load has been associated with inefficient neural processing (Barulli and Stern, 2013).

With respect to training effects, several studies have indicated that WM training leads to an increase in WM performance (Klingberg et al., 2002, 2005; Westerberg et al., 2007; Holmes et al., 2009, 2010; Thorell et al., 2009; Strobach et al., 2014). More importantly, WM training has been shown to improve performance in a broad range of other cognitive domains, such as executive control (Olesen et al., 2004; Klingberg et al., 2005; Westerberg et al., 2007; Thorell et al., 2009; Chein and Morrison, 2010; Brehmer et al., 2012; Strobach et al., 2014), episodic memory (Dahlin et al., 2008b; Lövdén et al., 2010; Richmond et al., 2011), and fluid intelligence (Klingberg et al., 2002; Olesen et al., 2004; Jaeggi et al., 2008, 2010; Rudebeck et al., 2012; Stephenson and Halpern, 2013; Au et al., 2015). Moreover, WM training is effective in older adults and has the potential to reduce age-related WM decline (Li et al., 2008; Schmiedek et al., 2010; Richmond et al., 2011; Brehmer et al., 2012; Buschkuehl et al., 2012; Heinzel et al., 2014b, 2016). Likewise, training of specific executive control processes, so-called process-based interventions, show similar beneficial effects in young and older adults (e.g., Li et al., 2008; Karbach and Kray, 2009; Brehmer et al., 2012; Zinke et al., 2012, see Karbach and Verhaeghen, 2014 for a review). Research on dual-task training is an important field in this research domain with promising effects on both training and transfer tasks (Liepelt et al., 2011; Strobach et al., 2012, 2015). Cognitive training research assumes similar mechanisms underlying training and transfer effects. Most WM training studies suppose that training improves executive control processes that are involved in the transformation and coordination of WM contents (Baddeley, 2003; Curtis and D’Esposito, 2003; Fuster, 2004). Likewise, many dual-task training studies assume that executive control processes involved in the coordination of the two component tasks (Meyer and Kieras, 1997; Szameitat et al., 2002; Stelzel et al., 2007) is optimized via training (Liepelt et al., 2011; Strobach et al., 2012, 2015) and that learning such stimulus-independent processes forms the basis for transfer effects rather than the mere improvement in the specific component tasks.

Importantly, it has been suggested that age-related deficits in dual-task performance (Lindenberger et al., 2000; Hartley, 2001; Verhaeghen and Cerella, 2002; Dubost et al., 2006; Göthe et al., 2007; Granacher et al., 2011) and corresponding changes in neural activation (Hartley et al., 2011; Chmielewski et al., 2014) may result from an underlying WM dysfunction (Awh et al., 2006; Gazzaley and Nobre, 2012).

Considering the effectiveness of WM and dual-task training programs to achieve both training and transfer effects in older adults as well as the overlapping constructs of WM and dual-task (Sala et al., 1995; Baddeley, 1996; Hegarty et al., 2000), we assume that improvements in dual-task performance reflected by a decrease in dual-task costs can be obtained by a WM training. According to notions of “neural transfer” (Dahlin et al., 2008a; Buschkuehl et al., 2012; Heinzel et al., 2014a), a training-related increase in neural efficiency of WM processing may facilitate executively demanding dual-task coordination due to an increased availability of neural resources related to dual-task coordination over and above modality-specific improvements in the component single-tasks.

To our knowledge, it has not been studied systematically if dual-task costs can be reduced by the training of a single n-back WM task in older adults. Thus, the present study aimed to contribute by answering the question whether this WM training leads to a transfer effect to dual-task performance. We trained older adults in a single n-back task with visually presented numerical stimuli (Cohen et al., 1997). The transfer dual-task consisted of a novel multimodal delayed match-to-sample paradigm that includes visual and auditory stimulus modalities. We hypothesized that the transfer from WM training effects to executive dual-task processes would improve performance in both stimulus modalities in the dual-task context, as measured by reduced dual-task costs for both tasks.

The results of the current study included older participants from a previously published training group that performed the n-back task during functional magnetic resonance imaging (fMRI) measurement pre and post-training (Heinzel et al., 2014a, 2016) as well as an unpublished control group. As reported in (Heinzel et al., 2014a, 2016), blood-oxygen-level-dependent (BOLD) signal in WM-related fronto-parietal regions was found to decrease in lower WM load after training in the training group, thus indicating a training-related increase in processing efficiency in WM (Lustig et al., 2009).

We investigated the hypothesis that training-related changes of BOLD response in literature-based WM-related regions of interest (ROIs) during one-back (low WM load) predict dual-task costs after training. According to previous research on neural correlates of both central executive components of WM (D’Esposito et al., 2000; Collette and Van der Linden, 2002; Baddeley, 2003; Mohr et al., 2006) and dual-task coordination (Goldberg et al., 1998; Herath, 2001; Loose et al., 2003; Schubert and Szameitat, 2003; Nebel et al., 2005; Yildiz and Beste, 2015), we expect that changes in BOLD response in dorsolateral prefrontal cortex (DLPFC) will be related to behavioral transfer effects to a dual-task.

## Materials and Methods

### Participants

Altogether, 38 older adults (range: 60–72 years) were recruited by announcements in local newspaper and the internet. In four participants the dual-task data was not correctly recorded due to technical failures during data acquisition. Therefore, the total sample consisted of 34 participants (see **Table 1**). Eighteen participants (11 females; mean ± SD age = 65.78 ± 3.04) were included in the training group and 16 participants (11 females, mean ± SD age = 65.00 ± 3.67) in a no-contact control group that was matched one by one to the training group according to age, sex, and education to ensure parallelization of the two groups. Thirteen participants of the training group in the current study also participated in fMRI sessions before and after the training program. Detailed fMRI results have been previously reported (Heinzel et al., 2014a, 2016). FMRI-analyses presented in the current paper specifically test the hypothesis that pre–post activation changes in a DLPFC ROI may predict results in a behavioral dual-task at T2. All participants were native German speakers, right-handed (Oldfield, 1971), had normal or corrected-to-normal vision, no psychopharmacological medication or history of any psychiatric disease, and achieved 27 or more points in the Mini Mental Status Examination (MMSE, Folstein et al., 1975). Written informed consent was obtained from each participant after the procedures had been explained. The study was approved by the local Ethics Committee of the Charité Universitätsmedizin Berlin, Germany and was conducted in accordance with the Declaration of Helsinki.

**Table 1:** Demographic variables.

	Training group *M (SD*)	Control group	Training vs. Control group *t*(32) (*p*)
Sex	7 males/11 females	5 males/11 females	χ^2^(34,1) = 0.216, *p* = 0.729
Age (years)	65.78 (3.04)	65.00 (3.67)	0.676 (0.504)
Education (years)	15.53 (2.76)	16.34 (3.63)	0.743 (0.463)
MMSE (points)	29.22 (1.17)	29.07 (0.99)	0.426 (0.673)

*MMSE, Mini Mental Status Examination.*

### Design and Procedure

At the beginning and the end of the training/waiting period, all participants completed both an n-back and a dual-task. Note that other results from the training group including neuropsychological tests and a Sternberg transfer task are reported elsewhere (Heinzel et al., 2016). The WM training was accomplished over a period of 4 weeks and contained 12 training session of an adaptive n-back training (approximately 45 min each). Training sessions took place on Mondays, Wednesdays, and Fridays in a quiet room at St Hedwig Hospital, Berlin, Germany. The control group was not contacted during this time. All tasks were presented with the software Presentation (Version 14.9; Neurobehavioral Systems).

#### n-Back Task and Adaptive Training

**FIGURE 1:**
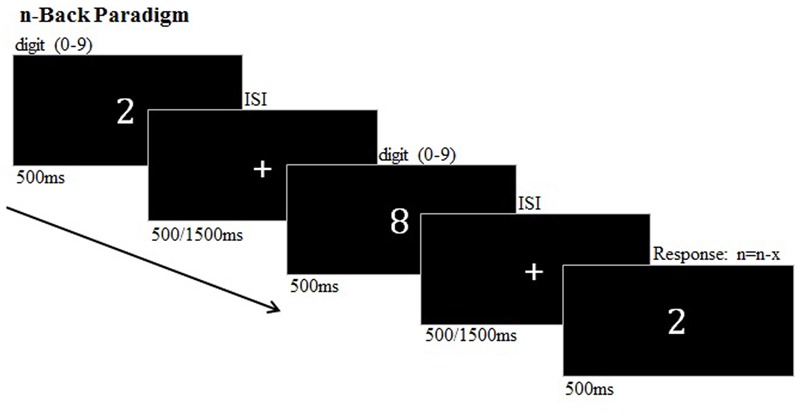
**n-back paradigm (example: two-back target)**.

The n-back task comprised two runs, each consisted of 16 blocks which were counterbalanced across subjects and presented in four pseudorandomized orders. Between the blocks, a white fixation cross was presented for 12 s. During the n-back paradigm a randomly assigned sequence of 16 numerical stimuli (0–9) was presented (Cohen et al., 1997) (**Figure 1**). The stimuli were presented separately in the center of a black screen for 500 ms. Two difficulty levels are induced by two different interstimulus intervals (ISIs) of 500 or 1500 ms (pseudorandomized between blocks). The subjects were required to indicate the re-occurrence of a number which has previously presented one, two, or three trials before (1-, 2-, 3-back) by a button press. During a zero-back condition, the participants were obliged to detect the number ‘0.’ The respective WM load condition (0-, 1-, 2-, and 3-back) was indicated by a cue 2 s before a block began. The n-back task lasted approximately 22 min.

The training group participated in an n-back training program over a period of 4 weeks with three sessions per week, resulting in 12 training sessions. Participants accomplished three runs of the n-back task in each training session, lasting approximately 45 min. Each run consisted of 12 blocks. At run 1 in session 1, all participants began the training with the difficulty level 1 (four blocks of zero-back, four-blocks of one-back, and four-blocks of two-back, at an ISI of 1500 ms). Difficulty level was individually adapted throughout all 12 training sessions in order to keep the task challenging during the entire training program (Doumas et al., 2009). The difficulty level of the task varied across training runs according to individual performance. Task difficulty was increased by introducing higher WM load levels and by shortening the ISI (Heinzel et al., 2014c). If a participant successfully completed one run with a hit rate of 80% or above within each block and with a false alarm rate below 15%, the next difficulty level was introduced in the following run. From level 1 to level 3, ISI was gradually decreased from 1500 to 500 ms in steps of 500 ms. At level 4, the next n-level was introduced (three-back), and zero-back was removed, i.e., participants completed 1-, 2-, and 3-back tasks. In addition, ISI was set back to 1500 ms. At level 7, four-back was introduced and one-back was removed.

#### Transfer Task

**FIGURE 2:**
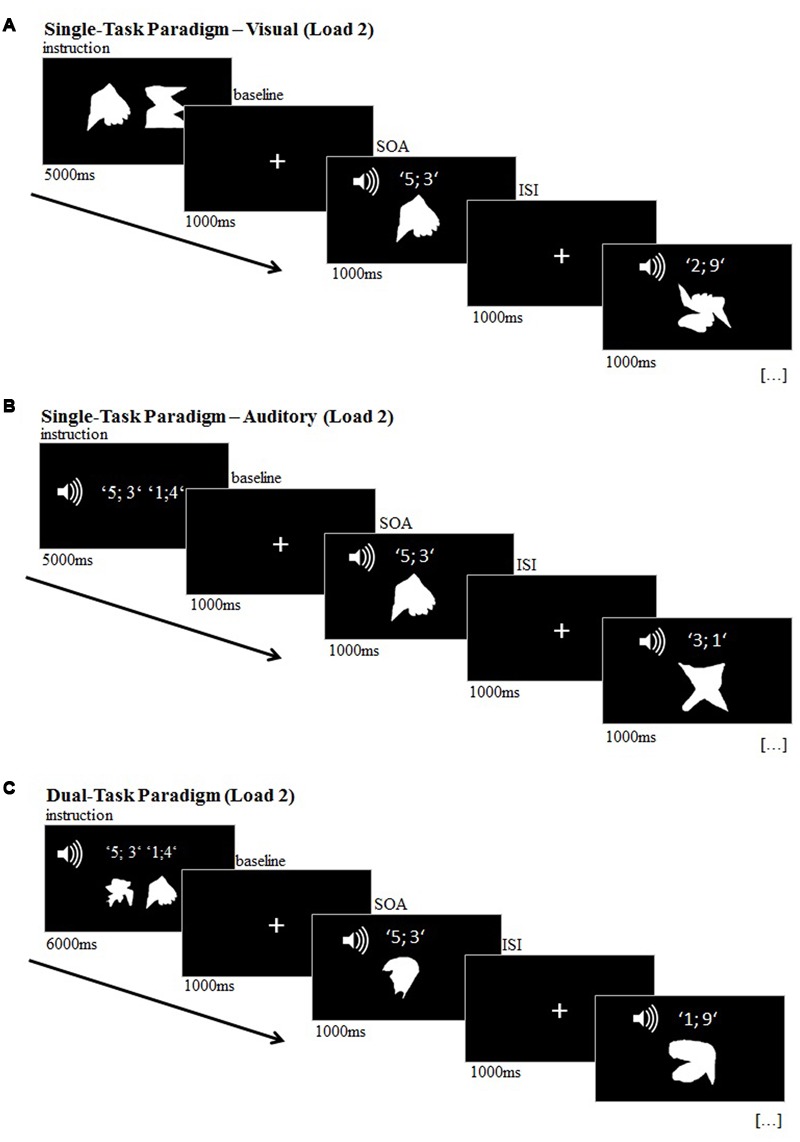
**Transfer task, illustrated for load 2 (‘difficult’); (A)** visual single-task; **(B)** auditory single-task; **(C)** dual-task.

The transfer dual-task consisted of a delayed match-to-sample paradigm in which the participants had to remember previously presented visual and/or auditory target stimuli. During the probe phase of the experiment, 16 visual and 16 auditory stimuli were presented successively, while one visual and one auditory stimulus was always presented simultaneously. In the single-task conditions, participants were instructed to attend to either the visual stimuli in the visual task (**Figure 2A**) and to auditory stimuli in the auditory task (**Figure 2B**). In the dual-task condition, participants had to attend to both visual and auditory stimuli (**Figure 2C**). There were two difficulty conditions for each task (memory load 1 and memory load 2) and each condition was presented twice in a pseudo-randomized order.

##### Visual and auditory single-tasks

**FIGURE 3:**
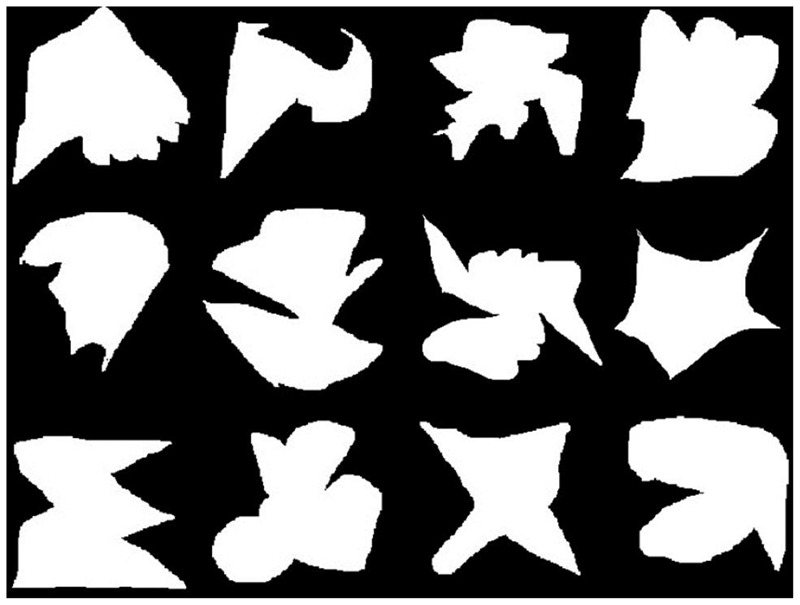
**Visual stimulus set: 12 meaningless shapes**.

The visual stimulus set consisted of 12 meaningless white shapes (**Figure 3**) and the auditory stimulus set consisted of 12 different pairs of digits, ranging from 0 to 9 (e.g., ‘4; 1’) and were presented by a female voice via speakers. In the encoding phase at the beginning of each block of the visual single-tasks, 1 (load 1: ‘easy’) or 2 (load 2: ‘difficult’) visual target stimuli were presented for 4000 ms (load 1) or 5000 ms (load 2), respectively. In the auditory single-task blocks, 1 (load 1) or 2 (load 2) number pairs were presented vocally as target stimuli. During this encoding phase, subjects were required to encode the target(s). Subsequently, in the probe phase of all conditions of the experiment, 16 visual and 16 auditory stimuli were presented randomly for 1000 ms each with ISIs of 1000 ms, while one visual and one auditory stimulus was always presented simultaneously. Each block included six target stimuli. Participants were requested to press the right mouse key on the laptop with the right index finger each time a target stimulus appeared. Thus, response modality was a motor response for all types of targets.

##### Dual-task

In the dual-task condition, 1 (load 1) or 2 (load 2) visual target stimuli and 1 or 2 auditory target stimuli were presented during the encoding phase of each block for 5000 ms (load 1) or 6000 ms (load 2). The probe phase in the dual-task conditions was identical to the single-task conditions, however, participants were required to attend to both visual and auditory stimuli at the same time. Each time one of the memorized targets matched a presented stimulus, the participant had to indicate the match by a button press.

### Performance and Dual-Task-Costs

The absolute task performance (percent correct) was calculated as the difference of the mean of hits minus false alarms (Eq. 1):

(1) hits−false alarms = absolute performance

The dual-task costs (relative dual-task performance) were calculated as the difference of the mean of single-task performance minus the mean of dual-task performance divided by the mean of single-task performance (Eq. 2):

(2) $$\frac{Performance Single Task-Performance Dual Task}{Performance Single Task}\cdot 100 = Dual Task Costs$$

Analyses of transfer effects to the dual-task were focused on dual-task costs because this measure defines dual-task performance in relation to and controlling for individual differences in single task performances. Therefore, dual-task costs are a more specific measure of executive control functions that are required to simultaneously perform two tasks and might be specifically sensitive for detecting age-related changes (Göthe et al., 2007).

### Analyses in fMRI Subgroup

#### MR Image Acquisition and Processing

A detailed description of the MR image acquisition and processing can be derived from (Heinzel et al., 2014a). 13 participants of the older training group of the current dual-task study also participated in pre- and post- fMRI-measurements during n-back as reported in (Heinzel et al., 2014a, 2016). In the beginning of each scanning procedure, one T1-weighted 3D pulse sequence was obtained. Functional data were obtained using a gradient echo echo-planar imaging (GE-EPI) pulse sequence (TR = 2000 ms, TE = 35 ms, flip angle = 80°, matrix size = 64 × 64, voxel size = 3.1 mm × 3.1 mm × 3.8 mm). 31 slices were acquired approximately axial to the bicommissural plane.

#### Estimation of BOLD Effect Sizes in n-Back

The WM experiment was analyzed within the framework of the general linear model (GLM). To this end, at the single subject level, we created design matrices comprising the experimental conditions of 0-, 1-, 2-, and 3-back as separate regressors of interest and all other experimental conditions (cue, button presses, and the six rigid body realignment parameters) as regressors of no interest. The GLM was fitted voxel-wise into the filtered time series using the restricted maximum likelihood algorithm as implemented in SPM8. We computed differential contrasts 1-back vs. 0-back, 2-back vs. 0-back, and 3-back vs. 0-back. Parameter estimates of BOLD response were extracted for seven literature-based ROIs (see Heinzel et al., 2014a for the procedure) which comprised the bilateral DLPFC, rostrate cingulate zone (RCZ), lateral premotor cortex (LPMC), and intraparietal sulcus (IPS). All ROIs combined define the WM network here. Change scores of n-back activity were calculated by subtracting parameter estimates at T2 from T1.

## Results

### WM Training

**FIGURE 4:**
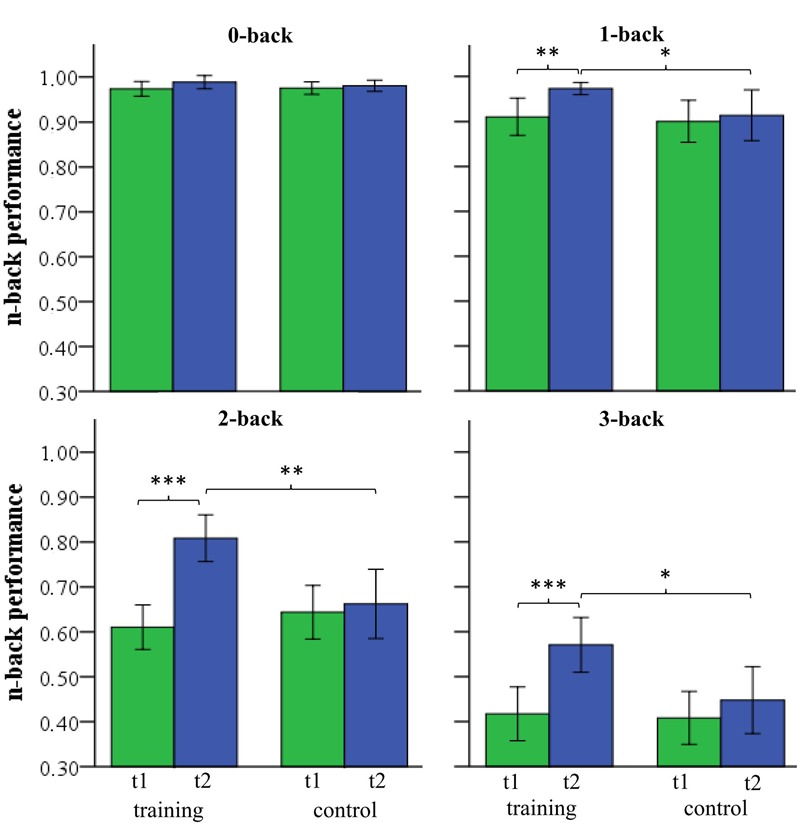
**n-back performance of the training and control group at pre-test (T1) and post-test (T2) for 0-, 1-, 2-, and 3-back (^∗^*p* < 0.05; ^∗∗^*p* < 0.01; ^∗∗∗^*p* < 0.001)**.

In order to assess WM training success a 2 (group) by 2 (time) by 4 (WM load) repeated-measures ANOVA was conducted (**Figure 4**). The ANOVA revealed significant interactions of the factors group by time by WM load (*F*_(3,30)_ = 7.309, *p* = 0.001, $\eta_{p}^{2}$ = 0.422) and group by time (*F*_(1,32)_ = 25.602, *p* < 0.001, $\eta_{p}^{2}$ = 0.444), as well as a significant main effect of time (*F*_(1,32)_ = 41.431, *p* < 0.001, $\eta_{p}^{2}$ = 0.564) and a non-significant main effect of group (*F*_(1,32)_ = 3.158, *p* = 0.085, $\eta_{p}^{2}$ = 0.090). *Post hoc* two-sample *t*-test revealed that both groups did not differ in any condition (0-, 1-, 2-, 3-back) at time T1 (**Table 2**). At time T2 both groups did not differ in the zero-back condition (*t*_(32)_ = 0.810, *p* = 0.424). Both groups differed significantly in the one-back (*t*_(32)_ = 2.159, *p* = 0.038), two-back (*t*_(32)_ = 3.203, *p* = 0.003), and three-back conditions (*t*_(32)_ = 2.578, *p* = 0.015). The control group did not show an improvement from T1 to T2 (all *p*’s > 0.11), whereas the training group improved significantly in WM performance from T1 to T2 for one-back (*t*_(15)_ = 3.400, *p* = 0.003), two-back (*t*_(15)_ = 7.368, *p* < 0.001), and three-back (*t*_(15)_ = 4.568, *p* < 0.001, see **Table 2**).

**Table 2:** Working memory performance.

	Training group			Control group			*t*-test (between-subject)	
Task	T1	T2	*t*-test (T1 to T2)	T1	T2	*t*-test (T1 to T2)	T1	T2
Zero-back	0.97 (0.04)	0.99 (0.03)	*t*_(15)_ = 1.515, *p* = 0.148	0.97 (0.03)	0.98 (0.03)	*t*_(17)_ = 0.794, *p* = 0.440	*t*_(32)_ = 0.003, *p* = 0.998	*t*_(32)_ = 0.810, *p* = 0.424
One-back	0.91 (0.09)	0.97 (0.03)	*t*_(15)_ = 3.400, *p* = 0.003	0.91 (0.09)	0.91 (0.11)	*t*_(17)_ = 0.131, *p* = 0.898	*t*_(32)_ = 0.022, *p* = 0.983	*t*_(32)_ = 2.159, *p* = 0.038
Two-back	0.61 (0.11)	0.81 (0.11)	*t*_(15)_ = 7.368, *p* < 0.001	0.65 (0.12)	0.66 (0.16)	*t*_(17)_ = 0.448, *p* = 0.661	*t*_(32)_ = -1.135, *p* = 0.265	*t*_(32)_ = 3.203, *p* = 0.003
Three-back	0.42 (0.13)	0.57 (0.13)	*t*_(15)_ = 4.568, *p* < 0.001	0.42 (0.12)	0.45 (0.15)	*t*_(17)_ = 1.670, *p* = 0.116	*t*_(32)_ = 0.048, *p* = 0.962	*t*_(32)_ = 2.578, *p* = 0.015

*Mean (SD); T1, pre-test; T2, post-test.*

### Single-Task Performance (Percent Correct)

Single-task performance of visual and auditory single-tasks are reported in **Table 3**.

**Table 3:** Single- and dual-task performance (in %).

Modality	Load	Training group				Control group			
		Single task		Dual task		Single task		Dual task	
		T1	T2	T1	T2	T1	T2	T1	T2
Visual	1	0.91 (0.15)	0.98 (0.04)	0.76 (0.22)	0.88 (0.18)	0.96 (0.05)	0.96 (0.06)	0.75 (0.34)	0.84 (0.21)
Visual	2	0.70 (0.23)	0.78 (0.17)	0.12 (0.26)	0.36 (0.27)	0.67 (0.20)	0.75 (0.23)	0.32 (0.28)	0.32 (0.31)
Auditory	1	0.96 (0.07)	0.98 (0.07)	0.77 (0.22)	0.93 (0.07)	0.97 (0.06)	0.99 (0.03)	0.77 (0.25)	0.82 (0.21)
Auditory	2	0.79 (0.17)	0.85 (0.17)	0.36 (0.28)	0.40 (0.24)	0.83 (0.16)	0.85 (0.19)	0.33 (0.29)	0.34 (0.23)

*Mean (SD); T1, pre-test; T2, post-test.*

#### Visual Single-Task Performance (Percent Correct)

A 2 (group) by 2 (time) by 2 (load) repeated-measures ANOVA showed no significant group by time by load interaction (*F*_(1,32)_ = 0.600, *p* = 0.444, $\eta_{p}^{2}$ = 0.018). Also, the group by time interaction (*F*_(1,32)_ = 0.500, *p* = 0.485, $\eta_{p}^{2}$ = 0.015) as well as all other interactions were not significant (all *p*’s > 0.32). A significant main effect of load (*F*_(1,32)_ = 69.595, *p* < 0.001, $\eta_{p}^{2}$ = 0.685) shows that performance decreased with increasing load. A significant main effect of time (*F*_(1,32)_ = 5.537, *p* = 0.025, $\eta_{p}^{2}$ = 0.147) indicates general improvement in visual task performance from T1 to T2. There was no significant effect of group (*F*_(1,32)_ = 0.030, *p* = 0.864, $\eta_{p}^{2}$ = 0.001).

#### Auditory Single-Task Performance (Percent Correct)

Comparable to the findings in visual single-task performance, a 2 (group) by 2 (time) by 2 (load) repeated-measures ANOVA in auditory task performance showed no significant group by time by load interaction (*F*_(1,32)_ = 0.196, *p* = 0.661, $\eta_{p}^{2}$ = 0.006). The group by time interaction (*F*_(1,32)_ = 0.508, *p* = 0.481, $\eta_{p}^{2}$ = 0.016) as well as all other interactions were not significant (all *p*’s > 0.44). A significant main effect of load (*F*_(1,32)_ = 42.558, *p* < 0.001, $\eta_{p}^{2}$ = 0.571) shows that performance decreased with increasing load. The main effect of time (*F*_(1,32)_ = 3.938, *p* = 0.056, $\eta_{p}^{2}$ = 0.110) was not significant. On a trend-level, this result may suggest a general improvement in auditory task performance from T1 to T2. There was no significant effect of group (*F*_(1,32)_ = 0.250, *p* = 0.620, $\eta_{p}^{2}$ = 0.008).

Taken together, the results of single-task analyses show that there is no transfer effect to any measure of single-task performance indicated by non-significant group by time interactions. This is also reflected by non-significant *post hoc* two-sample *t*-test (all *p*’s > 0.19).

### Absolute Dual-Task Performance (Percent Correct)

Mean values and standard deviations of absolute dual-task performance are reported in **Table 3**. A 2 (group) by 2 (time) by 2 (load) by 2 (modality) repeated-measures ANOVA showed no significant four-way interaction (*F*_(1,32)_ = 2.435, *p* = 0.129, $\eta_{p}^{2}$ = 0.071) and none of the three-way interactions was significant (all *p*’s > 22). A significant group by time interaction (*F*_(1,32)_ = 4.686, *p* = 0.038, $\eta_{p}^{2}$ = 0.128), indicated a training-related improvement in dual-task performance in the training group but not in the control group independently of load or modality. A significant main effect of time (*F*_(1,32)_ = 14.507, *p* = 0.001, $\eta_{p}^{2}$ = 0.312) shows a general improvement in dual-task performance from pre- to post-test. A significant main effect of load (*F*_(1,32)_ = 262.019, *p* < 0.001, $\eta_{p}^{2}$ = 0.891) reflects a generally lower performance at high load. No main effect of group was found (*F*_(1,32)_ = 0.031, *p* = 0.861, $\eta_{p}^{2}$ = 0.001). *Post hoc* two-sample *t*-test showed lower performance in the training group for visual targets during dual-task in the high load condition at T1 (*t*_(32)_ = 2.163, *p* = 0.038) and higher performance in the training group for auditory targets during dual-task in the low load condition at T2 (*t*_(32)_ = 2.074, *p* = 0.046). All other two-sample *t*-test were non-significant (all *p*’s > 0.46).

### Relative Dual-Task Performance (Dual-Task Costs)

**FIGURE 5:**
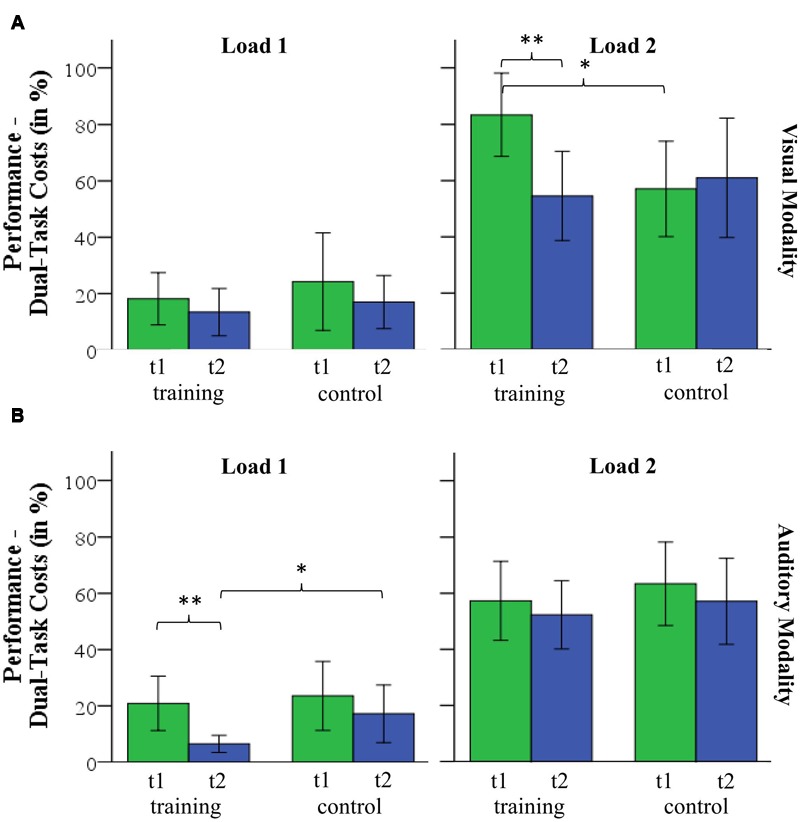
**Dual-task costs for load 1 and load 2 for (A)** visual and **(B)** auditory modality. ^∗^*p* < 0.05; ^∗∗^*p* < 0.01.

Mean dual-task costs and standard deviations as well as the results of the *post hoc* analyses are reported in **Table 4**. A 2 (group) by 2 (time) by 2 (load) by 2 (modality) repeated-measures ANOVA revealed a significant interaction of group by time by load by modality (*F*_(1,32)_ = 4.559, *p* = 0.041, $\eta_{p}^{2}$ = 0.125) and non-significant interactions of the factors group by time by modality (*F*_(1,32)_ = 0.587, *p* = 0.449, $\eta_{p}^{2}$ = 0.018) and group by time by load (*F*_(1,32)_ = 1.000, *p* = 0.325, $\eta_{p}^{2}$ = 0.030) and group by time (*F*_(1,32)_ = 3.066, *p* = 0.090, $\eta_{p}^{2}$ = 0.087). A significant main effect of time (*F*_(1,32)_ = 5.648, *p* = 0.024, $\eta_{p}^{2}$ = 0.150) indicates changes of dual-task cost from time T1 to T2. There was no significant effect of group (*F*_(1,32)_ = 0.007, *p* = 0.937, $\eta_{p}^{2}$ < 0.001). For the visual modality, *post hoc* two-sample *t*-test show that both groups differed significantly at T1 for load 2 (*t*_(32)_ = 2.564, *p* = 0.015). From T1 to T2, dual-task costs in the training group decreased from 83 to 54% (*t*_(17)_ = 3.531, *p* = 0.003) but did not change significantly in the control group (T1: 50%; T2: 59%, *t*_(15)_ = -0.541, *p* = 0.596, see **Figure 5A**). Both groups did not differ at T2 for load 2 (*t*_(32)_ = -0.302, *p* = 0.764). Within the auditory condition, *post hoc t*-test revealed that dual-task costs decreased in the training group from T1 (20%) to T2 (5%, *t*_(17)_ = 3.324, *p* = 0.004) but did not change in the control group (T1: 20%; T2: 17%, *t*_(17)_ = 0.405, *p* = 0.691) for load 1 (see **Figure 5B**). Both groups did not differ in load 1 at T1 (*t*_(32)_ = -0.035, *p* = 0.972) but at T2 (*t*_(32)_ = -2.415, *p* = 0.022). Within-subject comparisons can be found in **Table 4**. Dual-task costs for each condition are illustrated in **Figure 5**.

**Table 4:** Dual-task costs (relative performance in %).

Modality	Load	Training group			Control group			*t*-test (between subject)	
		T1	T2	*t*-test (T1 to T2)	T1	T2	*t*-test (T1 to T2)	T1	T2
Visual	1	16.55 (21.13)	10.67 (19.71)	*t*(17) = 0.897, *p* = 0.382	23.60 (35.11)	12.63 (22.24)	*t*(15) = 1.458, *p* = 0.166	*t*(32) = -0.719, *p* = 0.478	*t*(32) = -0.272, *p* = 0.787
Visual	2	83.37 (31.24)	54.33 (34.03)	*t*(17) = 3.531, *p* = 0.003	49.94 (44.34)	58.50 (46.07)	*t*(15) = -0.541, *p* = 0.596	*t*(32) = 2.564, *p* = 0.015	*t*(32) = -0.302, *p* = 0.764
Auditory	1	19.89 (21.52)	4.60 (8.00)	*t*(17) = 3.324, *p* = 0.004	20.19 (27.54)	17.20 (20.49)	*t*(15) = 0.405, *p* = 0.691	*t*(32) = -0.035, *p* = 0.972	*t*(32) = -2.415, *p* = 0.022
Auditory	2	53.39 (36.72)	52.33 (25.84)	*t*(17) = 0.098, *p* = 0.923	59.50 (37.32)	57.13 (30.65)	*t*(15) = 0.191, *p* = 0.851	*t*(32) = -0.481, *p* = 0.634	*t*(32) = -0.495, *p* = 0.624

*Mean (SD); T1, pre-test; T2, post-test.*

### Analyses in fMRI Subgroup

As reported in Heinzel et al. (2014a), there was a training-related reduction in BOLD-response during n-back in the WM network. This reduction was found to be specifically strong in the low WM load condition one-back, indicating an increase in neural efficiency. Here, we tested the hypothesis whether this training-related reduction in BOLD-response in the WM network and more specifically in DLPFC, can predict dual-task costs at post-test.

#### Individual Differences in the Training Group: Correlations with Dual-Task Costs

**FIGURE 6:**
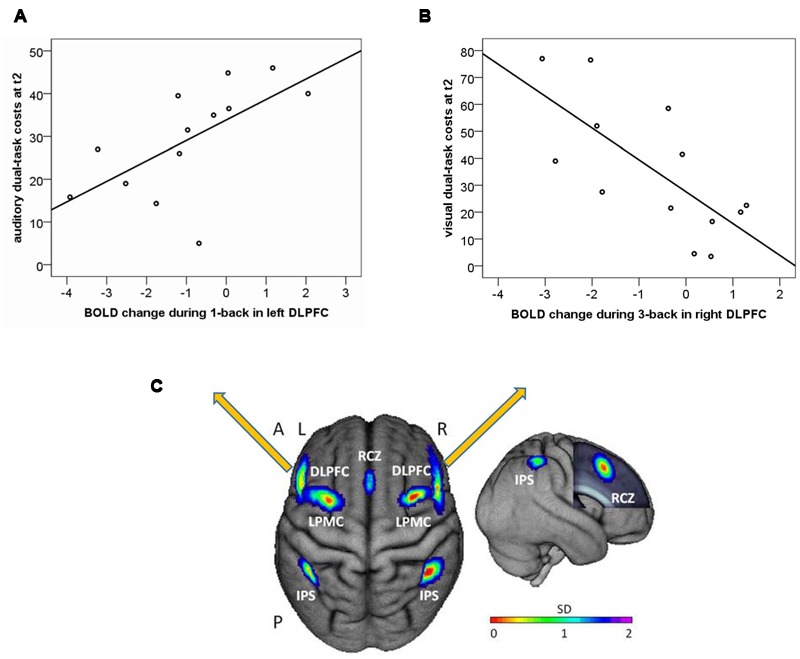
**Correlation of one-back BOLD change from T1 to T2 (arbitrary units) and auditory dual-task costs at post-test (T2) for (A)** left dorsolateral prefrontal cortex (DLPFC); and **(B)** right dorsolateral prefrontal cortex (DLPFC); **(C)** Location of literature-based probabilistic ROIs of the WM network. Left item: dorsal view of the ROIs overlaid onto the surface of the sample mean brain. Right item: right lateral view. The frontal lobe was cut to display the mid-sagittal ROI. RCZ, rostral cingulate zone; DLPFC, dorsolateral prefrontal cortex; LPMC, lateral premotor cortex; IPS, intraparietal sulcus; A, anterior; P, posterior; L, left; R, right; SD, standard deviation.

While no correlations between the entire WM network ROI (see **Figure 6C**) and dual-task costs were found (*p*’s > 0.24), analysis in left DLPFC revealed a significant correlation between training-related reduction in one-back activity and auditory dual-task costs at post-test (*r* = 0.625, *p* = 0.022), indicating lower dual-task costs in participants that showed a stronger reduction in one-back activity (**Figure 6A**). No correlations were found between right DLPFC and auditory dual-task costs (*r* = 0.297, *p* = 0.325). No significant correlations were found between DLPFC activation changes during one-back and visual dual-task costs (*p*’s > 0.35). However, exploratory analyses in 2- and 3-back revealed a significant negative correlation between changes in right DLPFC activity during three-back and visual dual-task costs at post-test (*r* = -0.711, *p* = 0.006, see **Figure 6B**), indicating an increase in three-back activity could have been beneficial for visual dual-task performance.

## Discussion

In the present study, we aimed to investigate the influence of WM training on dual-task costs in a novel delayed match-to-sample dual-task. The results indicate that 12 sessions of numerical n-back training can improve the performance in the trained task. Moreover, we found a transfer to the performance in a dual-task. The transfer was reflected by a reduction of dual-task costs in the ‘easy’ auditory condition in the training but not the control group. Further, we found a reduction of dual-task costs in the ‘difficult’ visual condition. No training-associated changes in single task performance were found. This is in line with previous research (e.g., Liepelt et al., 2011; Bonato et al., 2013), indicating that measures of dual-task coordination seem to be more sensitive to both subtle cognitive deficits and training-related changes. An additional analysis of a subsample within the training group of this dual-task study that also participated in a previously published fMRI study (Heinzel et al., 2014a, 2016), revealed that a reduction in one-back activity from pre- to post-test in the left DLPFC predicted dual-task costs in the auditory task at post-test. Additional exploratory analyses indicated that changes in three-back activity in right DLPFC were associated with dual-task costs in the visual task at post-test. Thus, taken together, fMRI results may suggest that a training-related reduction in DLPFC activity during low WM load as well as an increase during high WM load might support dual-task performance.

### Transfer Effects

The assumption that numerical n-back training can reduce dual-task costs in an untrained transfer task has been partly confirmed. Unexpectedly, modality-specific transfer effects were found to be dependent on the task demand. While transfer to the auditory task occurred in the ‘easy’ condition, transfer to the visual task was found in the ‘difficult’ condition. Especially in the training group, performance in the high load condition of the visual task was lower compared to the auditory task at T1, possibly indicating a task-prioritization (Stelzel et al., 2009) biased toward the auditory task. A diminished difference between visual and auditory performance during dual task after training, supports the notion of a shift toward a more efficient dual-task coordination, accompanied by the ability to focus on both component tasks.

Since a transfer effect to both modalities of the dual-task and no transfer to the single-tasks was found, the WM training in this study might have led to an improved modality-independent executive control that was not restricted to the modality of the internal stimulus representation of the trained n-back task (verbal representation of numbers). Therefore, n-back training may facilitate the coordination of two simultaneous tasks as suggested by models and empirical work on central executive processes (Morris and Jones, 1990; Baddeley, 2000; Collette and Van der Linden, 2002). These central executive processes are thought to comprise an attentional control system that governs other WM subsystems including information storage and rehearsal (Baddeley, 2000), and can be divided into separate subfunctions [e.g., updating, inhibition, shifting, dual task coordination (Miyake et al., 2000; Collette and Van der Linden, 2002)].

The results of the current study add an important piece of information to the current cognitive training literature, as we could show that improvements in dual-task performance do not necessarily require an explicit dual-task training if the applied single task training includes executive control processes that are continuously demanding on the WM system. This notion may be derived from studies comparing different training and transfer paradigms including single choice reaction tasks (Strobach et al., 2012, 2015) and studies comparing adaptive to non-adaptive training regimes (Brehmer et al., 2011, 2012). In fact, improvements in dual-task performance in older adults may be enlarged if crucial components are specifically trained (Strobach et al., 2012) and task demand of the training task is adaptively increased according to individual performance (Brehmer et al., 2012).

Please note that the term “transfer effects” that is used in the current study refers to a relatively narrow concept of transfer [“near transfer,” see taxonomy by Noack et al. (2009)], as training and transfer tasks share several process components. On the other hand, the transfer task (dual-task) also differed in crucial aspects from the training task (e.g., attending to two modalities instead of one, use of different stimuli, complex delayed match to sample instead of updating task). Furthermore, no improvements were found in the control group, neither in the training nor in the transfer task. Thus, we are confident that improvements in the transfer task were not just due to familiarity effects in the training task. Since we only used a no-contact control group in the current study, however, we cannot rule out an additional influence of familiarity effects.

The investigation of neural mechanisms underlying “far transfer,” e.g., transfer to tasks that include motor coordination such as postural control tasks could be an interesting focus for future research in older adults. In fact, recent work on cognitive-motor dual-tasking in older adults (for review see Boisgontier et al., 2013) has indicated that training-related cognitive improvements might also facilitate postural control performance, a strong predictor for risk of falls (Beauchet et al., 2009).

### Analyses within the fMRI Subgroup

As an additional analysis in a subsample (*N* = 13 training participants), we investigated whether a WM training-related increase in neural efficiency in n-back can predict dual-task costs at post-test. Our hypothesis of a DLPFC-modulated transfer to auditory dual-task costs was confirmed by the current data. A reduction in left DLPFC activation during one-back from pre- to post-test in the training group may indicate an increase in neural efficiency in WM, which could have facilitated auditory task performance in a multimodal dual-task. However, as re-test effects cannot be excluded, this cannot be directly derived from the current investigation and requires confirmation in further studies.

Against our hypothesis, the magnitude of visual dual-task costs was not predicted by a DLPFC activity reduction during one-back. However, exploratory analyses revealed that those participants showing a training-related increase in three-back activity in right DLPFC also showed the lowest visual dual-task costs at post-test. Previous research on DLPFC functions suggests its role in higher order executive control such as chunking (Bor et al., 2003, 2004; Owen et al., 2005), maintaining (Courtney, 1998; Nee et al., 2013), updating, and manipulating of information (Owen, 1997; Roth et al., 2006; Barbey et al., 2013). Thus, a more efficient processing in, e.g., chunking may have been beneficial for dual-task performance in the present study in terms of a reduction in auditory dual-task demands by an improvement in the conjunction of stimulus information. Potentially, an overlapping internal stimulus representation (a verbal “code,” Wickens, 2002) of the training task and the auditory transfer task may have additionally supported transfer effects within this modality domain.

Hypothetically, the modality-specific findings in DLPFC may relate to a predominantly left lateralized processing of verbal information in WM as compared to predominantly right lateralized processing of visuospatial information (Smith and Jonides, 1999). More specifically, previous research indicates that right hemispheric DLPFC might be predominantly involved in controlling visuospatial WM, whereas left hemispheric DLPFC would mainly control verbal WM (Baddeley, 2003). The association between right-hemispheric DLPFC activity changes during three-back and transfer to visual dual-task could relate to a training-related improvement in neural capacity (Barulli and Stern, 2013) in some subjects as discussed in the model of training-related neural adaptations by Lustig et al. (2009). This potential capacity adaptation may have facilitated the visuospatial task performance within the dual-task condition. However, due to the small sample size of the current pilot study and limitations in study design, these interpretations are only preliminary.

### General Limitations and Perspectives

There are several limitations that need to be taken into account when interpreting the results of this study. First, we did not measure fMRI during the dual-task. Thus, we cannot make reliable statements about neuronal effects during dual-task processing in this study. The pre–post fMRI results reported here are based on a relatively small sample and no pre–post fMRI data from the dual-task control group was available. Therefore, any kind of conclusion should be made with restraints as re-test effects cannot be excluded. Further limitations are the lower dual-task performance for visual targets in the high load condition of the training compared to the control group at pre-test and very high performance in both groups in single tasks at low load. Thus, the possibility for further improvement was restricted in the easy conditions of the single tasks. Future studies should include larger samples and compare different age groups such as children and young adults. This would increase the significance in terms of allowing extensive assumptions about cognitive plasticity across the life span. Also, for reasons of feasibility, we only included a no-contact control group. Therefore, social interaction or other unspecific effects associated with the training procedure might have influenced results in the training group and the influence familiarity/practice effects cannot be ruled out. In future studies, an active control group should be included in the study design. Another limitation is related to the sustainability of the results. The current experimental design does not allow any assumptions about long-lasting training or transfer effects. Future studies should take account of implementing follow-up measures.

## Conclusion

In the current study, a 4-week WM training intervention (single n-back task) in a sample of older adults was associated with an increase in WM performance in the training group but not in an untrained control group. Furthermore, a transfer effect from single-task n-back training to dual-task performance was reported in older adults for the first time. Additional analyses of training-related changes of BOLD response during WM processing in relation to post-training dual-task performance provide preliminary evidence for neural underpinnings of this transfer effect. The findings support the notion of a training-related increase in neural efficiency, as indicated by a reduced activity in DLPFC during one-back performance that may have facilitated the reduction of auditory dual-task costs at post-test after training. Additional analyses in right DLPFC during performance of three-back suggest that increased WM capacity might support performance in a visuospatial dual-task condition. Findings may indicate a training-related improvement of central executive functioning.

## Author Contributions

SH and MR designed the study. SH programmed the experiments. SH and JR supervised and conducted the experiments. SH, JR, CS, and MR analyzed the data. SH, JR, CS, and MR wrote the paper.

## Conflict of Interest Statement

The authors declare that the research was conducted in the absence of any commercial or financial relationships that could be construed as a potential conflict of interest.
